# A systematic screening assay identifies efficient small guide RNAs for CRISPR activation

**DOI:** 10.3389/fbioe.2025.1336313

**Published:** 2025-01-23

**Authors:** Elin Arvidsson, Diana Duarte Lobo, Ermelinda Sabarese, Fabio Duarte, Rui Jorge Nobre, Luis Quintino, Cecilia Lundberg

**Affiliations:** ^1^ CNS Gene Therapy, Department of Experimental Medical Sciences, Lund University, Lund, Sweden; ^2^ CNC - Center for Neuroscience and Cell Biology of Coimbra, University of Coimbra, Coimbra, Portugal; ^3^ Institute for Interdisciplinary Research, University of Coimbra, Coimbra, Portugal; ^4^ ViraVector – Viral Vector for Gene Transfer Core Facility, University of Coimbra, Coimbra, Portugal

**Keywords:** CRISPRa, gene activation, gene therapy, *Tfeb*, *Adam17*, *Sirt1*

## Abstract

CRISPR-mediated gene activation (CRISPRa) encompasses a growing field of biotechnological approaches with exciting implications for gene therapy. However, there is a lack of experimental validation tools for selecting efficient sgRNAs for downstream applications. Here, we present a screening assay capable of identifying efficient single- and double sgRNAs through fluorescence quantification *in vitro*. In addition, we provide a tailored Golden Gate cloning workflow for streamlined incorporation of selected sgRNA candidates into lentiviral (LVs) or adeno-associated viral vectors (AAVs). The overall workflow was validated using therapeutically relevant genes for neurodegenerative diseases, including *Tfeb*, *Adam17*, and *Sirt1*. The most efficient sgRNAs also demonstrated activation of endogenous gene expression at mRNA level. Correlation analysis of gene activation relative to sgRNA binding site distance to transcription start-site or nearby transcription factor binding sites failed to detect common characteristics influencing gene activation in the selected promoter regions. This data demonstrates the potential of the screening assay to identify functionally efficient sgRNA candidates across multiple genes along with streamlined cloning of viral vectors and may assist in accelerating future developments of CRISPRa-focused applications.

## 1 Introduction

The discovery of clustered regularly interspaced short palindromic repeats (CRISPR) DNA segments and CRISPR-associated proteins (Cas), known as CRISPR/Cas, has revolutionized biomedical research (Katti et al., 2022). There is an ever-growing number of CRISPR/Cas systems engineered for genome editing. Interestingly, most of the CRISPR/Cas systems adapted for biotechnological purposes operate similarly. A Cas protein containing a small guide RNA (sgRNA) targets a nucleic acid sequence complementary to the binding sequence of the sgRNA and adjacent to a protospacer adjacent motif (PAM). The Cas/sgRNA complex then performs a double stranded break of the targeted region.

This modularity enables the adaptation of CRISPR/Cas systems to mediate programable genetic functions beyond gene editing. For example, CRISPR/Cas systems can perform specific epigenetic modulation, gene inhibition and gene activation (CRISPRa) (Kampmann, 2020). To adapt CRISPR/Cas systems for CRISPRa, the Cas catalytic domains are silenced and effector domains from transcription factors are fused to the silenced (or, “dead”) Cas9 protein or bound to sgRNA via RNA loops. The Cas/sgRNA/effector complex targets a genomic DNA sequence and activates gene expression when bound upstream of transcriptional start sites (TSS) (Gilbert et al., 2014).

Diverse CRISPRa systems have been previously described in the literature and among them, CRISPRa-VPR (Chavez et al., 2015) has been widely used. VPR, due to its reproducible gene activation, now serves as a benchmark for testing new CRISPRa systems (Chavez et al., 2016; Zhang et al., 2018). This effector domain consists of a fusion protein of VP64, p65 and Rta transcriptional effector domains. In a previous collaboration with the Church lab, our lab aided in the validation of a smaller version of the VPR domain (Vora et al., 2018) that together with a compact Cas could be used with viral vectors for gene therapy applications *in vivo*. The compact CRISPRa system was further engineered (MiniCas9V2) for applications in neurons and demonstrated activation of therapeutically relevant genes in the brain (Maria et al., 2020).

As gene therapies typically require the use of viral vectors as delivery tools, specifically lentiviral vectors (LV) and adeno associated vectors (AAV), the CRISPRa components need to be sufficiently small to be packaged into an LV or AAV. Accordingly, several factors need to be considered. First, a miniature Cas protein (Vora et al., 2018; Xu et al., 2021) is optimal. Second, a compact effector domain with proven capability of gene activation *in vivo*, such as SAM or VPR (Liu et al., 2021), should be tested. Lastly, the lowest possible number of sgRNAs should be used.

Presently, there are several Cas proteins and compact effector domains available for viral vector usage *in vivo* (Zhou et al., 2018; Colasante et al., 2020). However, there is a lack of *in vitro* screening methods for selection of functional sgRNAs to minimize the number of sgRNA needed for *in vivo* CRISPRa studies. In addition to reducing the size of the genetic payload that needs to be packaged into viral vectors, decreasing the number of necessary sgRNAs also minimizes the possible off target effects (Casas-Mollano et al., 2020; Schoger et al., 2020). Therefore, validating and selecting a minimal number of functional sgRNAs will further support the design of novel and sophisticated CRISPRa strategies. Without functional screening of sgRNA, up to four sgRNAs may be needed to ensure activation of target genes *in vivo* (Maria et al., 2020).

To optimize sgRNA for CRISPRa *in vivo*, it is important to have a fast, simple, and systematic screening assay. Ideally, such an assay should be incorporated in a streamlined cloning workflow to generate LV and AAV quickly and efficiently. With these features in mind, a screening assay was designed using plasmid transfection and fluorescent protein readouts *in vitro*, coupling the plasmids used to a tailored Golden Gate cloning workflow to facilitate incorporation into LV and AAV. The screening assay was validated using therapeutically relevant genes for neurodegenerative diseases such as Alzheimer’s Disease, Parkinson’s Disease and Machado-Joseph Disease. Specifically, a disintegrin and metalloprotease 17 (*Adam17*) (Mockett et al., 2017), transcription factor EB (*Tfeb*) (Rai et al., 2021) and Sirtuin (*Sirt1*) (Cunha-Santos et al., 2016) were targeted for activation.

The CRISPRa screening identified sgRNAs that led to robust activation of *Adam17*, *Tfeb* and *Sirt1*. The top sgRNA candidates were further validated for endogenous gene activation through transfection in the Neuro2A mouse neuroblastoma cell line. Several sgRNAs identified in the screening were able to induce endogenous gene expression. Taken together, these results suggest that the CRISPRa screening assay can identify single or dual sgRNA capable of activation of multiple therapeutically relevant genes in a simple and time-efficient manner. The described screening assay, together with the cloning workflow, should accelerate the development of novel CRISPRa applications for biomedical purposes.

## 2 Materials and methods

### 2.1 In silico small guide RNA design

To generate sgRNAs, 22-nt spacer sequences were designed to target a 500–600 bp region immediately upstream of the *Tfeb*, *Adam17*, and *Sirt1* transcription start site (TSS). Spacer sequences were identified using the University of California Santa Cruz (UCSC) genome browser (www.genome-euro.ucsc.edu) and the CHOPCHOP web tool (Labun et al., 2019) and validated for template-strand targeting using the Benchling online setup tool (Benchling, 2023) (Supplementary Figure 1). Seven binding sites were selected for each promoter, as this allowed single and dual sgRNA screening in a 96-well format. A 22-nt scrambled spacer (Scr) that did not target any sequence in the mouse genome, verified via BLAST, was generated as a control sgRNA used in all subsequent experiments. A standard *S. aureus*-compatible RNA scaffold with a terminator sequence (gtt​tta​gta​ctc​tgg​aaa​cag​aat​cta​cta​aaa​caa​ggc​aaa​atg​ccg​tgt​tta​tct​cgt​caa​ctt​gtt​ggc​gag​att​ttt​tt) was added downstream of the sgRNA spacer sequence. The sequences used to generate nonpalindromic overhangs for assembly were previously described by the Barrick laboratory (Barrick, 2021). All visualization of DNA segments and assembly of constructs was performed *in silico* using SnapGene (Dotmatics).

### 2.2 TOPO cloning

Constructs containing individual sgRNA segments, scrambled filler segments or destination blocks were designed as GeneArt™ Strings™ DNA fragments (ThermoFisher Scientific). The gene strings consisted of an expression cassette, dubbed DPL0, that contained a hU6-promoter, sgRNA binding site, *S. aureus* sgRNA scaffold and poly-T terminator sequence. The DPL0 cassette was flanked by a 4 bp overhang sequence and *Esp3I* recognition sites to generate non-palindromic nucleotide overhangs upon restriction. Filler blocks were designed as random 400 nt segments similarly flanked by a 4 bp overhang sequence along with *Esp3I* restriction sites to ensure that the downstream Golden Gate cassettes were similar in size. In addition, a L3G0 cassette consisting of multiple Golden Gate destination sites and standard multiple cloning sites was also designed for downstream assembly of the selected sgRNAs cassettes. Gene strings were inserted into a pCR^®^II-TOPO^®^ vector using the Zero Blunt™ TOPO™ PCR Cloning Kit according to the manufacturer’s instructions (Invitrogen) to generate pDPL0, pScramble and pLG0 constructs, respectively.

### 2.3 Gateway cloning

Gateway 4.1.2 assembly was used to generate pHG.EF1a.MiniCas9V2, pHG.mTfeb.TdTomato, pHG.mAdam17.TdTomato and pBG.mSirt1.TdTomato reporter constructs. All plasmids containing the promoter regions were ordered via gene synthesis as Gateway-compatible donor plasmids (ThermoFisher Scientific). Promoter sequence and expression cassette from donor constructs pEntry.P4P1R.EF1a and pEntry.MiniCas9V2 (Maria et al., 2020) were assembled in the pHG destination vector (Quintino et al., 2013) to generate the pHG.EF1a.MiniCas9V2 construct. For the TdTomato reporters, donor vectors pEntry.P4P1.mTfeb, pEntry.P4P1R.mAdam17 or pEntry.P4P1.mSirt1, along with pEntry.TdTomato, were assembled in pHG or pBG destination vectors. Reactions were performed using the Gateway™ LR Clonase™ II Enzyme mix (Invitrogen) according to manufacturer’s directions.

### 2.4 Golden Gate cloning

Expression cassettes were excised from the pDPL0 vector through *Esp3I-*digestion and ligated in tandem using T4 ligase (20 U/µl, New England Biolabs) for assembly into the *Esp3I*-digested pLG0 vector backbone. Inserts and backbone were added to the reaction mix at a 1:1 insert:vector ratio using 20 fmol of each plasmid and supplemented with 50 mM ATP and 100 mM DTT. Assembly was performed at a 20 µL final reaction volume. Thermal cycling was programmed for 25 cycles of digestion at 37°C for 2 min and ligation at 16°C for 5 min, followed by *Esp3I* digestion at 60°C for 10 min and finalization by heat inactivation at 80°C for 20 min. This cloning was performed in accordance with protocols previously established by Haynes and Barrett (Haynes and Barrett, 2013).

### 2.5 Standard molecular cloning

Assembly cloning was used to insert expression cassettes into AAV- or lentiviral compatible vectors. pLG0-constructs and the pAAV-U6-mAtct1-Sa acceptor vector (Vora et al., 2018) were digested with *NotI-MfeI*, while the pHG.EF1a.m.RFP acceptor vector was digested with *SpeI*. Digested products were separated on a 1% agarose gel. Bands corresponding to the expression cassette and digested acceptor vector, respectively, were excised and purified using the GeneJET Gel Extraction Kit (ThermoFisher Scientific) or the NucleoSpin Gel and PCR Clean-up Kit (Macherey-Nagel) according to the manufacturer’s instructions. Purified DNA concentrations were quantified against a GeneRuler 1 kb Plus DNA ladder (ThermoFisher Scientific) and ligation was performed at a 3:1 insert:vector ratio using Anza™ T4 DNA Ligase Master Mix (ThermoFisher Scientific) supplemented with 10 mM ATP. The ligation reaction was performed according to manufacturer’s instructions.

### 2.6 Sequencing

Finished constructs were Sanger sequenced through the Eurofins TubeSeq service (Eurofins Genomics). M13-forward and reverse universal primers were used to sequence the pDPL0 and pLG0 constructs.

### 2.7 Transformation

Bacterial transformation was performed in One Shot™ TOP10 (Invitrogen) for pDPL0 and pLG0 or Stbl3™ competent bacteria (Invitrogen) for the LV and AAV plasmid backbones, respectively. Reactions were performed according to manufacturer’s instructions, using 1 µL of ligated plasmid per reaction. One hundred microlitres of transformation mix was plated on antibiotic-supplemented LB agar plates and incubated overnight at 32°C or 37°C, respectively.

### 2.8 DNA extraction

Colonies from culture plates were selected and grown in 5 mL antibiotic-supplemented LB medium overnight (16 h). DNA was extracted using the QIAprep Spin Miniprep Kit (QIAGEN, Germany). Control restriction was performed using standard methods and correct clones were selected and grown overnight in 250 mL antibiotic-supplemented LB medium. The correct bacterial cultures were processed the following day using the NucleoBond Xtra Midi kit for transfection-grade plasmid DNA (Macherey-Nagel) according to the manufacturer’s instructions. Purified DNA was resuspended in molecular grade water to prevent downstream cloning inefficiency due to the presence of EDTA in standard resuspension buffer, especially when performing Golden Gate reactions.

### 2.9 Cell culturing and transfection for CRISPRa screening assay

Tissue culture plates were incubated with sterile water containing 0.002% Poly-L-Ornithine Solution (PLO) (Merck) overnight, washed twice with sterile water or PBS and left to dry fully in a ventilated hood before cell seeding. HEK 293T cells used in this study were obtained from the American Type Culture Collection cell biology bank (CRL-3216). HEK 293T cells were maintained in Dulbecco’s Modified Eagle Medium (DMEM) (Invitrogen), supplemented with 10% fetal bovine serum (FBS) (Cytiva) and 1% Penicillin-Streptomycin (P/S) (Invitrogen), were seeded at a density of 6000 cells per well. For transfection, pHG.EF1a.BFP, pHG.EF1a.TdTomato carrying the promoter of the gene of interest, pHG.EF1a.MiniCas9V2 and pDPL0 sgRNA constructs were resuspended in PBS at a 1:0.1:2:1 ratio to a total DNA content of 200 ng/well. Polyethyleneimine 25,000 (PEI) (1 mg/mL) was added to the plasmid mixture at a 5:1 PEI:DNA ratio and incubated for 15 min at RT before addition to the cell culture. Transfected cultures were incubated at 37°C for 48 h before media was aspirated. Cells were fixed with 4% PFA for 10–15 min at RT before being washed twice with PBS. The fixed cultures were quantified as described in the fluorescent analysis section below.

### 2.10 Gene expression assay

Neuro2A cells were maintained in culture with DMEM (Sigma-Aldrich) supplemented with 10% FBS and 1% P/S. Cells were seeded at a density of 300,000 cells/well in 6-well culture plates. Twenty-four hours later, transfection was performed using PEI MAX 40,000 (1 mg/mL) at 7.5:1 PEI:DNA ratio. pHG.EF1a.MiniCas9V2 was added along with the respective sgRNA candidates at a 2:1 (:1) ratio to a total of 600 ng DNA per well. The transfection mixture was vortexed and incubated for 15 min at RT before being added to the cell culture.

Fourty-eight hours later, cells were harvested and processed using the NucleoSpin RNA kit (Macherey-Nagel) or the PureLink RNA Mini Kit (ThermoFisher Scientific) according to manufacturer’s instructions. cDNA was synthesized from 1 µg of RNA per sample using the iScript cDNA Synthesis Kit (Bio-Rad) or iScript Reverse Transcription Supermix for RT-qPCR (Bio-Rad) according to manufacturer’s instructions. Quantitative real-time-PCR reactions were prepared as follows. For *Tfeb* and *Adam17*, reactions were set up at 10 µL reaction volume using LightCycler 480 SYBR Green Master Mix (Roche) and performed using the LightCycler 480 Real-Time PCR Instrument (Roche). The amplification protocol was performed as follows: pre-incubation at 95°C for 5 min, followed by 45 cycles of amplification including denaturation at 95°C for 10 s, followed by annealing at 60°C for 10 s, and extension at 72°C for 10 s. The melting curve was performed at 65°C and increased at a ramp rate of 4.8 °C/s up to 95°C with a hold time of 5 min. For *Sirt1*, quantitative real-time PCR was carried out at 20 µL reaction volume and performed in CFX96 Touch Real-Time PCR Detection System (Bio-Rad). The amplification protocol started with denaturation step at 95°C for 30 s, followed by 40 cycles of two steps: denaturation at 95°C for 5 s and extension/annealing at 60°C for 15 s. The melting curve was performed at 65°C for 5 s, and up to 95°C with an increment of 0.5°C. Results were analyzed in terms of mRNA quantification relative to control samples and determined by the Pfaffl method (Pfaffl, 2001). Values were analyzed using standard delta-delta-Ct calculations.

The following primers were used to amplify targets of interest: *Tfeb* (forward: ATC​CAG​AAG​CGA​GAG​CTA​AC, reverse: ATT​CCC​AGC​TCC​TTG​ATC​C), *Adam17* (forward: GTG​GCT​CTC​AAC​TCT​GTA​ACT​C, reverse: TTT​ACA​GCA​CTT​GGC​TTT​GTT​T), *Sirt1* (forward: AGC​GGC​TTG​AGG​GTA​ATC​A, reverse: ACT​GCC​ACA​GGA​ACT​AGA​GGA), *SaCas9* (forward: CTG​CTG​AAC​AAC​CCC​TTC​AAC, reverse: TTG​CTG​ATT​CTG​CCC​TTG​CC) and *Hprt* (forward: CTT​CCT​CCT​CAG​ACC​GCT​TT, reverse TCA​TCG​CTA​ATC​ACG​ACG​CT).

### 2.11 Fluorescence analysis

For *Tfeb* and *Adam17*, promoter activation was estimated though fluorescence quantification using a Plate RUNNER HD (TROPHOS). Alignment was performed using the Align software and fluorescent images of TagBFP2 (381/445 nm) and TdTomato (554/581 nm) expression were acquired using the Goelan software. Image analysis was performed using ImageJ (NIH). The total TdTomato fluorescence per plate was normalized by the total TagBFP2 fluorescence to adjust for transfection efficiencies. The mean TdTomato/TagBFP2 ratio of scramble samples was used as a baseline for subsequent calculations.

For *Sirt1*, Operetta CLS (PerkinElmer) was used for microplate fluorescence quantification. In brief, TagBFP2 cells were identified and TdTomato fluorescence in these cells was subsequently measured. The average sum per well of TdTomato fluorescence per cell was normalized by the average sum per well of TagBFP2 fluorescence per cell. The mean TdTomato/TagBFP2 ratio of Scr samples was used as baseline for subsequent calculations.

### 2.12 Synergy estimation

Effect-based methodology was used to assess synergy or antagonism between sgRNA. The significance of sgRNA interaction was first determined using two-way ANOVA (Slinker, 1998). For each sgRNA combination, the activation of Scr (control), activation of sgRNA A, activation of sgRNA B, and activation of sgRNA A + B were evaluated together. The null hypothesis was that activation of sgRNA A was independent from activation with sgRNA B. Whenever there was a statistically significant interaction effect between activation of sgRNA A and sgRNA B, the null hypothesis was rejected. In other words, the activation was most likely a result of a significant interaction between sgRNA A and sgRNA B. To determine if the nature of the interaction between sgRNA A and sgRNA B was positive (synergy) or negative (antagonism), the two-way ANOVA interaction results were analyzed together with a Combination Index (CI), which, due to the nature of the data, was calculated using a response additivity model (Foucquier and Guedj, 2015). The expected additive effect, Expected activation (A + B), was calculated by adding the activation of sgRNA A and activation of sgRNA B, as shown by the following equation: $E\;act\;\left(A+B\right)=act\;sgRNA\;A+act\;sgRNA\;B$ . The CI was calculated by dividing the Expected activation of sgRNA A and sgRNA B by the observed activation of combining sgRNA A and sgRNA B, as shown by the following equation: $CI=\frac{E\;act\left(A+B\right)}{act\;\left(A.B\right)}$ . In synergistic sgRNA interactions CI < 1, in additive or independent interactions CI = 1 and in antagonistic interactions CI > 1. By determining if sgRNA A and sgRNA B had a significant interaction and using the CI to estimate if this interaction was positive or negative, it was possible to infer significant synergistic or antagonistic interactions between sgRNA combinations.

### 2.13 Transcription factor binding site analysis

The promoter sequences of mouse *Tfeb*, *Adam17*, and *Sirt1* used to select sgRNA were analyzed using CiiiDER (Gearing et al., 2019) to predict transcription factor binding sites (TFBS). A stringency of 1 was used to select only TFBS with 100% homology to the promoter sequences. A TFBS cluster was defined as a DNA sequence where there are ≥10 TFBS overlapping or within 10 base pairs distance of each other.

### 2.14 Statistics

Statistical analysis was performed in GraphPad Prism 9 (Dotmatics). For cell culture experiments, experimental samples were compared against a scrambled control construct using multiple comparison ordinary One-Way ANOVA. Dunnett’s *post hoc* analysis was performed, using the Scr + dCas9 condition as control for comparison. Two-way ANOVA was used to calculate significant interaction scores. Histograms are presented as mean ± SEM.

Correlation analysis was performed to determine whether screening assay activation rates were influenced by the genomic landscape at the promoter region. First, activation rates of each sgRNA were analysed in relation to distance from the TSS. Additionally, activation rates of individual or dual sgRNAs were correlated to the binding site proximity to the closest TFBS cluster, and finally to the number of TFBS overlapping the sgRNA binding site, within 50 bp and within 100 bp. Whenever the data indicated two groups, a Welch’s t-test (not assuming equal standard deviations) was performed. Whenever the data indicated multiple groups, Dunnett’s *post hoc* analysis was performed. XY graphs are presented as mean activation plotted against distance from TSS, distance to nearest TFBS cluster, number of overlapping TFBS, number of TFBS within 50 bp and 100 bp, respectively. Each experiment was repeated at least twice.

## 3 Results

### 3.1 DPL0-L3G0 cloning workflow allows efficient and seamless assembly of multiple sgRNA combinations

**FIGURE 1:**
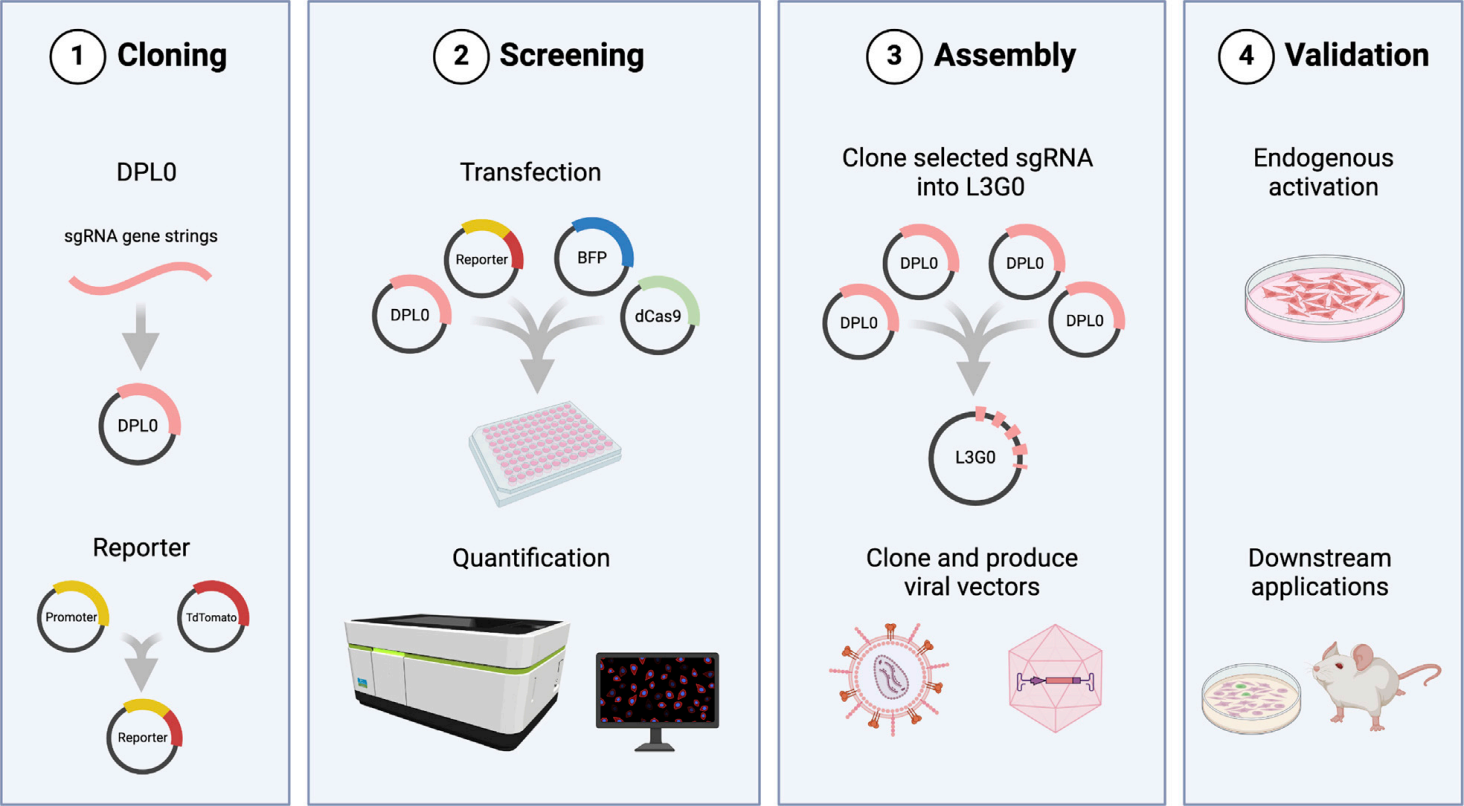
Overview of the cloning workflow and screening assay. 1) DPL0 cassettes containing candidate sgRNA sequences are cloned into TOPO vectors. The reporter plasmid consists of TdTomato driven by the promoter sequence for the gene of interest. 2) pDPL0 plasmids are transfected in combination with dCas9 plasmid, reporter, and BFP for normalization of fluorescence expression. Levels of TdTomato expression are normalized by BFP and quantified to identify transcriptionally efficient sgRNA combinations. 3) Selected cassettes are assembled in pLG0 constructs and can be subcloned into LV or AAV, according to research needs. 4) Final validation of sgRNA as plasmids or through viral vectors may be performed in culture or *in vivo*. Created in BioRender. Arvidsson, E. (2025) https://BioRender.com/a94y700.

**FIGURE 2:**
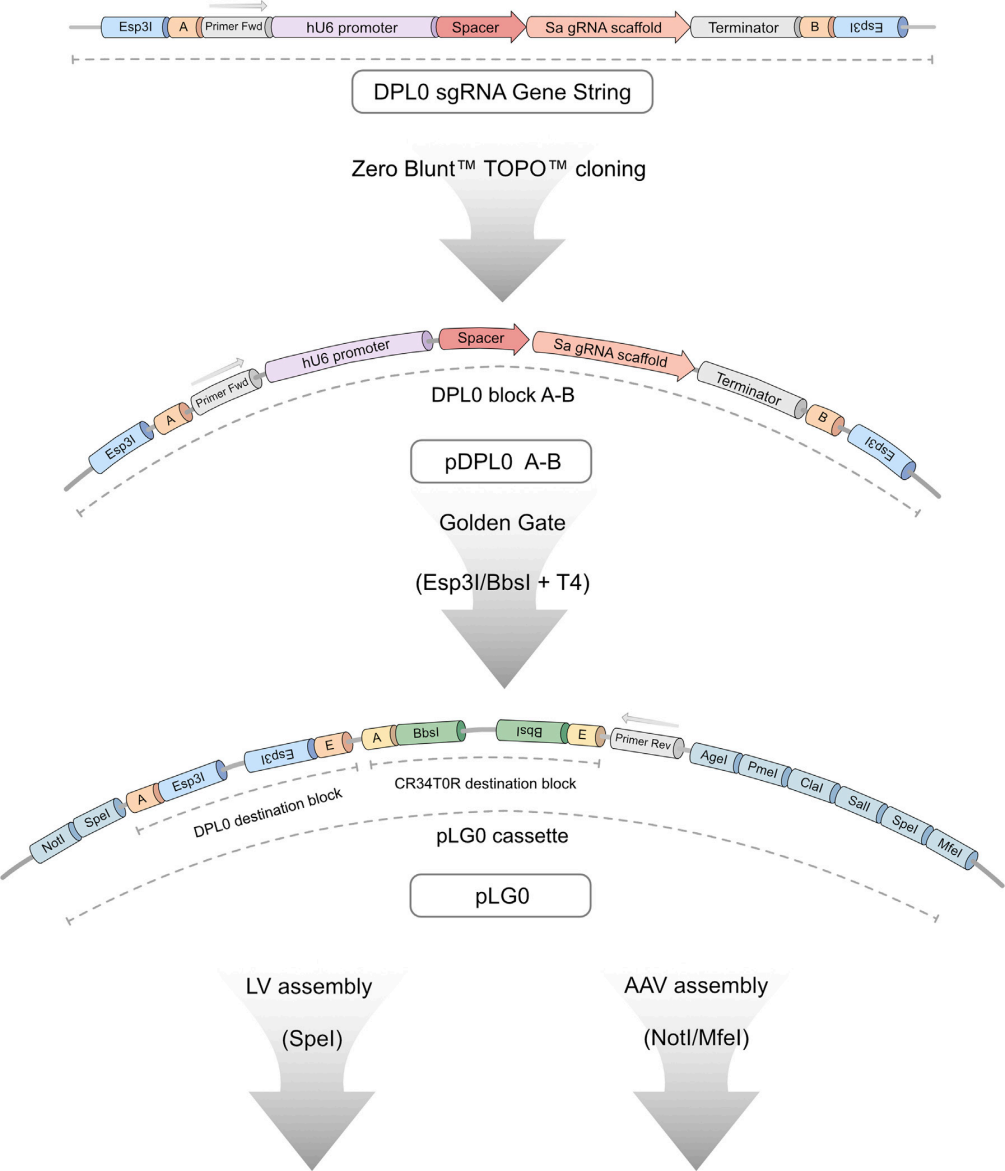
Detailed overview of L3G0 cloning workflow. All sgRNA candidates were ordered as gene strings consisting of a DPL0 cassette containing a human U6 promoter, spacer or binding site, SaCas9 RNA scaffold, and downstream terminator sequence. The expression cassette is flanked by Esp3I-restriction target sites, generating 4-bp overhang sequences compatible with ligation of four separate DPL0 blocks in tandem through Golden Gate cloning. Ligated cassettes are introduced into destination blocks on the pLG0 construct that contain Esp3I-compatible sites (DPL0 destination) and BbsI-compatible sites (CR34T0R destination). This design allows seamless assembly of up to eight sgRNA candidates within a single vector. When in pLG0, primer binding sites allow for screening using colony PCR. The LG0 cassette provides a multitude of additional restriction sites, allowing for downstream applications such as subcloning into LV- and AAV-compatible constructs.

The purpose of this study was to create an experimental screening system for sgRNA used for CRISPRa applications requiring viral vectors. Moreover, to make it truly universal in usage, the screening assay was coupled to a cloning workflow (Figure 1). This enables a streamlined validation process, from plasmid design to functional assessment of endogenous gene activation. The initial task involved establishing a robust and flexible cloning methodology. This tailored Golden Gate workflow, named Lund Efficient sgRNA Omnibus (L3G0) cloning, was designed in-house to provide a time- and labour-efficient strategy for subcloning multiple relevant sgRNA combinations into a single pLG0 plasmid that could then be transferred to plasmids containing LV or AAV backbones, according to specific research needs (Figure 2).

CRISPRa-compatible sgRNA binding sequences were selected using online bioinformatic tools such as Benchling and CHOPCHOP (Benchling, 2023; Labun et al., 2019) to target the 500–600 base pair promoter region immediately upstream of the TSS. This region was selected as previous studies have highlighted this genomic window as suitable for CRISPRa sgRNA targeting (Gilbert et al., 2014; Konermann et al., 2015). As *Tfeb*, *Adam17* and *Sirt1* were selected for proof-of-concept validation, the mouse *Tfeb*, mouse *Adam17* and mouse *Sirt1* promoter regions were used. In total, seven sgRNA candidate binding sequences for each gene of interest were selected for insertion into DPL0 expression cassettes subsequent screening analysis (Supplementary Figure 1).

### 3.2 CRISPRa screening assay determines experimental activation of promoters through fluorescence quantification

The CRISPRa screening assay was designed to experimentally estimate the activation efficiency of sgRNA identified using bioinformatic tools. The screening assay has four components: pDPL0 plasmids containing a sgRNA candidate, a plasmid expressing MiniCas9V2 (Maria et al., 2020), a reporter plasmid expressing TdTomato under the control of the promoter regions of interest, and a plasmid expressing TagBFP2 to be used as transfection control. The assay was performed by transfecting these four types of plasmids into HEK 293T-cells and assessing fluorescence 48 h after transfection. TagBFP2 expression was used as a transfection normalizer and the TdTomato expression in each condition was then compared to the mean ratio of a scramble (Scr) sgRNA control. Representative fluorescence images obtained using the *Sirt1* screening assay can be found in Supplementary Figure 2.

**FIGURE 3:**
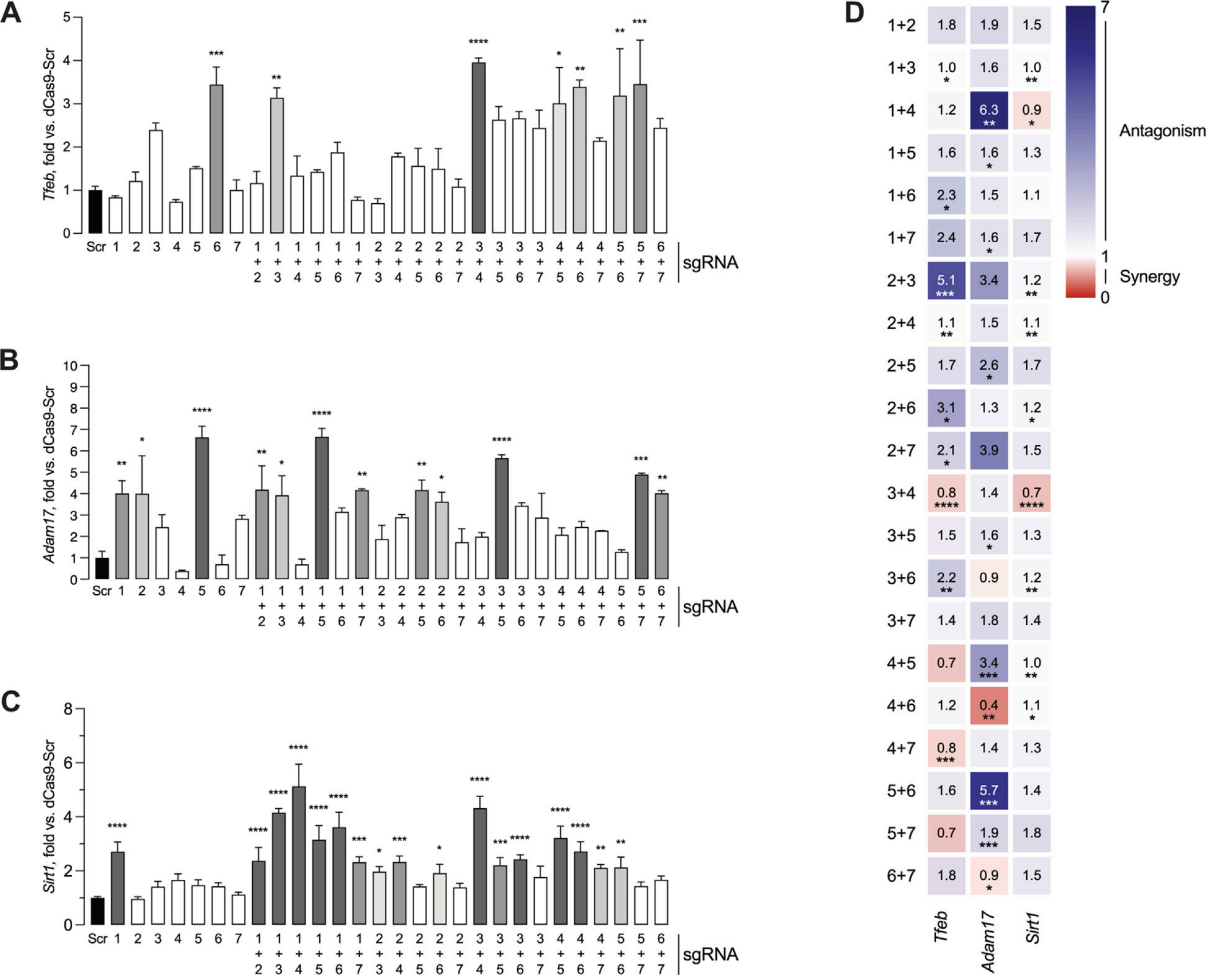
Screening assay identifies sgRNAs capable of activating transcription from *Tfeb*, *Adam17* and *Sirt1* promoters. HEK 293T cells were transfected with pDPL0 expressing sgRNA, reporter constructs expressing TdTomato, MiniCas9V2 and BFP plasmid. Forty-eight hours after transfection, the cells were fixed and the TdTomato and BFP fluorescence was measured. **(A)** Activation of *Tfeb* expression by single or dual sgRNA combinations. **(B)** Activation of *Adam17* expression by single or dual sgRNA combinations. **(C)** Activation of *Sirt1* expression by single or dual sgRNA combinations. All values are presented as mean ± SEM and normalized against a Scr control sample (left, black). For the histograms, bars with higher statistical significance are marked in darker shades of grey. **(D)** Calculation of synergy or antagonism for dual sgRNA combinations using two-way ANOVA interaction scores and Combination Index. Scr- Scramble. *P ≤ 0.05, **P ≤ 0.01, ***P ≤ 0.001, ****P ≤ 0.0001.

One-way ANOVA demonstrated statistically significant differences in mouse *Tfeb* promoter activation (Figure 3A) between sgRNA groups (F (28,58) = 6.34, p < 0.0001). Post hoc analysis with Dunnett test using the Scr condition as control showed that sgRNA 6 (3.45 ± 0.40 fold), sgRNA 1 + 3 (3.15 ± 0.23 fold), sgRNA 3 + 4 (3.96 ± 0.10 fold), sgRNA 4 + 5 (3.02 ± 0.83 fold), sgRNA 4 + 6 (3.40 ± 0.16 fold), sgRNA 5 + 6 (3.19 ± 1.09 fold) and sgRNA 5 + 7 (3.46 ± 1.02 fold) led to significant increases in TdTomato levels, indicating that these seven respective conditions resulted in robust mouse *Tfeb* promoter activation.

Similarly, one-way ANOVA showed statistically significant differences in mouse *Adam17* promoter activation (Figure 3B) between groups (F (28,58) = 8.2, p < 0.001). In the case of mouse *Adam17* sgRNA candidates, Dunnett *post hoc* test identified significantly increased promoter activation in a total of twelve combinations, including sgRNA1 (4.01 ± 0.59 fold), sgRNA 2 (4.00 ± 1.78 fold), sgRNA 5 (6.64 ± 0.52 fold), sgRNA 1 + 2 (4.19 ± 1.12 fold), sgRNA 1 + 3 (3.93 ± 0.91 fold), sgRNA 1 + 5 (6.66 ± 0.39 fold), sgRNA 1 + 7 (4.15 ± 0.06 fold), sgRNA 2 + 5 (4.16 ± 0.48 fold), sgRNA 2 + 6 (3.63 ± 0.45 fold), sgRNA 3 + 5 (5.66 ± 0.16 fold), sgRNA 5 + 7 (4.90 ± 0.06 fold), and sgRNA 6 + 7 (4.03 ± 0.11 fold).

In the case of mouse *Sirt1*, one-way ANOVA indicated statistically significant differences in mouse *Sirt1* promoter activation between groups (F (28,58) = 30.5, p < 0.0001, Figure 3C). Dunnett’s *post hoc* test indicated increased promoter activation in seventeen conditions, namely, sgRNA1 (2.7 ± 0.21 fold), sgRNA 1 + 2 (2.37 ± 0.29 fold), sgRNA 1 + 3 (4.16 ± 0.09 fold), sgRNA 1 + 4 (5.13 ± 0.47 fold), sgRNA 1 + 5, (3.15 ± 0.30 fold), sgRNA 1 + 6 (3.62 ± 0.32 fold), sgRNA 1 + 7 (2.3 ± 0.11 fold), sgRNA 2 + 3 (1.97 ± 0.10 fold), sgRNA 2 + 4 (2.33 ± 0.12 fold), sgRNA 2 + 6 (1.91 ± 0.19 fold), sgRNA 3 + 4 (4.32 ± 25 fold), sgRNA 3 + 5 (2.20 ± 0.16 fold), sgRNA 3 + 6 (2.42 ± 0.10 fold), sgRNA 4 + 5 (3.20 ± 0.25 fold), sgRNA 4 + 6 (2.72 ± 0.21 fold), sgRNA 4 + 7 (2.10 ± 0.07 fold) and sgRNA 5 + 6 (2.13 ± 0.22 fold).

Although it was expected that not all single sgRNA or dual sgRNA screened would consistently lead to increased promoter activity, the number of single or dual sgRNA combinations capable of significant gene activation varied between the different promoter regions, ranging from seven for *Tfeb* to seventeen for *Sirt1*. Additionally, varying combinations of sgRNA led to notably higher levels of activation. These findings suggest that efficient sgRNA binding and gene activation are promoter specific.

To further investigate possible interactions between sgRNA in the target promoters, significant synergistic or antagonistic activation effects were calculated for the different sgRNA combinations in all the genes tested (Figure 3D). In the case of *Tfeb*, combinations of sgRNA 1 + 6, sgRNA 2 + 3, sgRNA 2 + 4, sgRNA 2 + 6 and sgRNA 3 + 6 displayed an antagonistic interaction. In contrast, combinations of sgRNA 3 + 4 and sgRNA 4 + 7 had a synergistic interaction. For *Adam17*, sgRNA 1 + 4, sgRNA 1 + 5, sgRNA 1 + 7, sgRNA 2 + 5, sgRNA 3 + 5, sgRNA 4 + 5 and sgRNA 5 + 6 had an antagonistic interaction, while sgRNA 4 + 6 and sgRNA 6 + 7 exhibited a synergistic interaction. Finally, in the case of *Sirt1*, sgRNA 2 + 3, sgRNA 2 + 4, sgRNA 2 + 6, sgRNA 2 + 7, sgRNA 3 + 6, sgRNA 4 + 5, and sgRNA 4 + 6 had an antagonistic interaction, whereas sgRNA 1 + 4 and 3 + 4 had a synergistic interaction.

### 3.3 Correlation between sgRNA activation and promoter characteristics

sgRNA activation data generated from the screening assay was then correlated to the genomic context of each promoter of interest. First, the correlation between sgRNA activation and distance from the TSS was assessed. In addition, the correlation between sgRNA activation and number of transcription factor binding sites (TFBS) present within the promoter sequences was also determined.

The number of TFBS with 100% sequence homology present within the 600 bp promoter sequences was predicted using CiiiDER (Gearing et al., 2019). This prediction tool identified 251 TFBS present within the *Tfeb* promoter sequence, 71 TFBS present within the *Adam17* promoter sequence and 145 TFBS present within the *Sirt1* promoter sequence, respectively. Moreover, the number of TFBS clustered in the promoter sequences was analyzed. We considered a TFBS cluster as a promoter sequence containing ten or more TFBS overlapping or within 10 base pairs distance of one another. It was possible to identify two TFBS clusters present within the *Tfeb* promoter sequence, three TFBS clusters present within the *Adam17* promoter sequence and four TFBS clusters present within the *Sirt1* promoter sequence, respectively.

**FIGURE 4:**
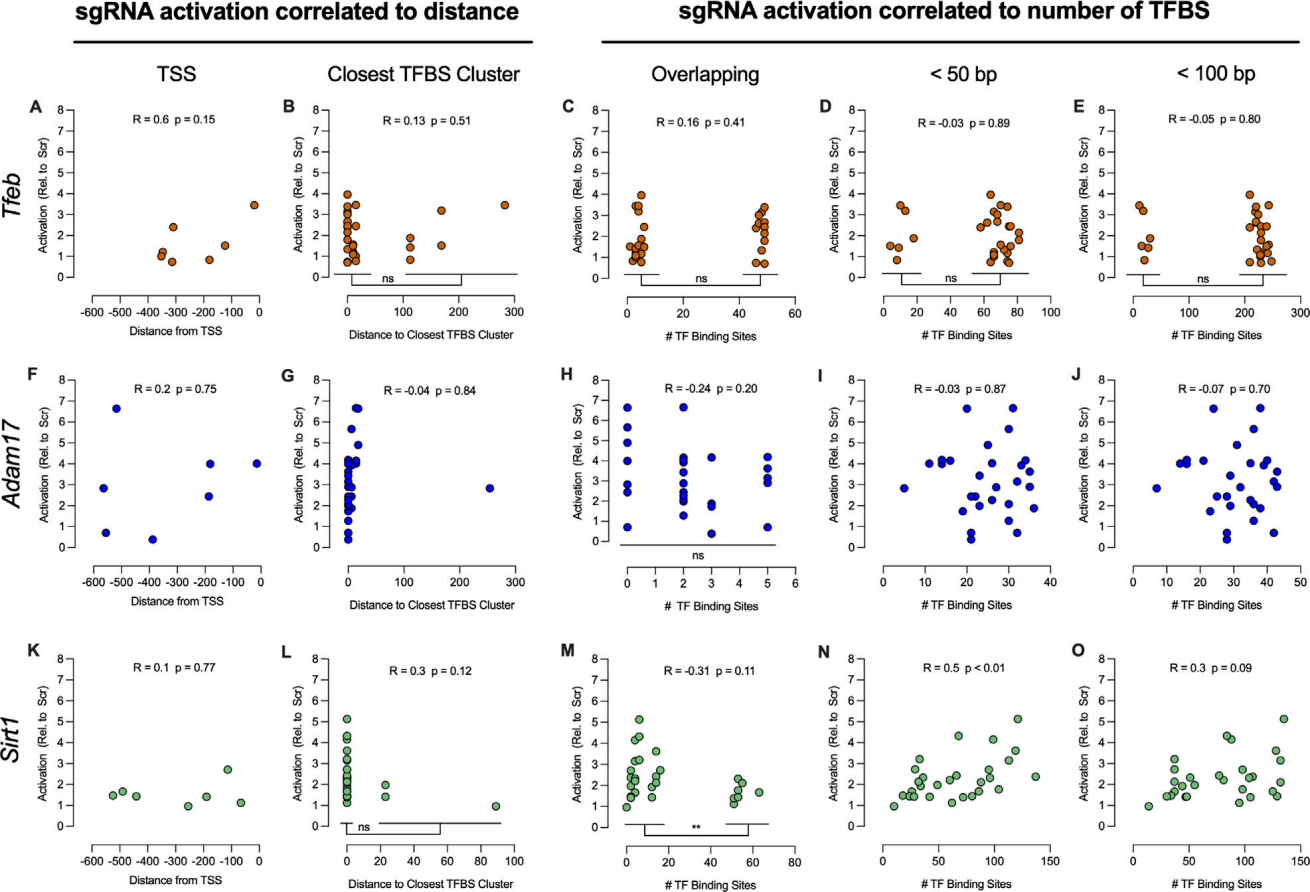
Correlation of sgRNA activation to TSS and TFBS present in 600 bp promoter sequences. Gene activation of seven respective sgRNA was correlated to the relative distance of the sgRNA binding sites from the TSS of *Tfeb* **(A)**, *Adam17* **(F)** and *Sirt1* **(K)** respectively. Gene activation relative to distance of the sgRNA binding sites to TFBS clusters was compared for *Tfeb* **(B)**, *Adam17* **(G)** and *Sirt1* **(L)**, respectively. Analysis of gene activation *versus* the number of overlapping TFBS, number of TFBS within 50 bp, or number of TFBS within 100 bp of the sgRNA binding sites of *Tfeb* **(C–E)**, *Adam17* **(H–J)**, *and Sirt1* **(M–O)** was also performed. For multiple comparisons, **P ≤ 0.01. ns-not significant.

First, sgRNA activation and distance from TSS or distance to closest TFBS cluster (Figures 4A, B, F, G, K, L) was compared. No correlation between sgRNA activation and distance from TSS was observed. Similarly, there was no correlation between sgRNA activation and distance to closest TFBS cluster. Whenever the data points separated into distinct groups such as activation distance to closest TFBS cluster for *Tfeb* and *Sirt1* (Figures 4B, L), the mean activation of the resulting groups was compared using Welch’s t-test. Both *Tfeb* (t (7.6) = 0.05, p = 0.96) and *Sirt1* (t (4.4) = 2.68, p = 0.05) showed no significant differences of sgRNA activation between groups.

The sgRNA activation and number of TFBS overlapping the sgRNA binding sequence, within 50 bp and within 100 bp of the sgRNA binding sequence, were also compared. In the case of *Tfeb* (Figures 4C–E), no correlation was observed. Although the activation data formed two clear groups in all *Tfeb* comparisons, no differences in activation were observed between groups when using Welch’s t-test: overlapping TFBS (t (25.52) = 0.8, p = 0.43), within 50 bp (t (7.63) = 0.04, p = 0.97) and within 100 bp (t (7.63) = 0.04, p = 0.97).

For *Adam17* (Figures 4H–J), no correlation between sgRNA activation and number of TFBS overlapping the sgRNA binding sequence, within 50 bp and within 100 bp respectively, was observed. Moreover, in the analysis of activation vs overlapping TFBS, no differences between groups were observed when using One-Way ANOVA analysis (F (3, 24) = 0.9, p = 0.455).

For *Sirt1*, no correlation between activation and distance to TSS or proximity to TFBS cluster was observed (Figures 4K, L). Furthermore, no correlation between activation and number of TFBS overlapping sgRNA binding sequence was observed (Figure 4M). However, the data indicated two distinct activation groups, based on the number of overlapping TFBS. Comparison of activation between these two groups using Welch’s t-test (t (25.2) = 2.9, p < 0.01) indicated significant differences between groups. Presence of more than 50 TFBS overlapping the sgRNA binding sequence resulted in significantly lower activation rates. Significant correlation (r (28) = 0.51, p < 0.01) was observed when comparing activation and number of TFBS within 50 bp of sgRNA binding sites (Figure 4N). When comparing activation and number of TFBS within 100 bp of sgRNA binding sites, no correlation was observed (Figure 4O).

In summary, assessment of activation *versus* distance to TSS or TFBS clusters did not show any correlation in the promoter regions of interest. Moreover, no correlation was observed between *Tfeb* and *Adam17* activation and number of TFBS close to sgRNA binding sequences. Interestingly, it was possible to determine that 50 or more TFBS overlapping the sgRNA binding sequence impaired *Sirt1* activation, whereas a higher number of TFBS within 50 bp lead to increased activation. Overall, correlation data failed to highlight common features of the promoter region that influence sgRNA activation.

### 3.4 Subcloning sgRNA into viral vector-compatible constructs

After determining sgRNA candidates capable of robust activation, the selected multiple sgRNA cassettes present in the pDPL0 plasmids were assembled into the pLG0 plasmid (Figure 2), as part of the L3G0 cloning workflow. This plasmid contains the LG0 cassette, consisting of two independent Golden Gate destination blocks surrounded by multiple cloning sites that were tailored to in-house LV- and AAV-compatible plasmids. The Golden Gate DPL0 destination block present in the pLG0 plasmid uses Esp3I for inserting sgRNA cassettes present in pDPL0 plasmids, whereas the CR34T0R destination block uses BbsI to insert sgRNA-compatible cassettes. These two Golden Gate destination cassettes were designed for seamless tandem insertion of up to eight sgRNA cassettes within a single vector. More sgRNA cassettes can be added by designing more overhangs within the DPL0 and CR34T0R blocks (Potapov et al., 2018) or inserting additional pre-assembled DPL0 or CR34T0R groups of sgRNA cassettes in the downstream multiple cloning site region. Even with both destination blocks in use, the LG0 cassette reaches a size of 3.4 kb, well below the packaging limit of an AAV. In the final part of the L3G0 cloning workflow, the multiple sgRNA cassettes present within the LG0 cassette are subcloned into compatible LV or AAV. For the following experiments involving endogenous gene activation, we resorted to plasmid transfection of separate DPL0 constructs into cell cultures.

### 3.5 Selected sgRNA candidates lead to increased endogenous gene activation

The next step was to validate the ability of the single sgRNA or dual sgRNA combinations to activate endogenous gene expression. pDPL0 plasmids carrying selected sgRNA were transfected together with MiniCas9V2 in Neuro2A cell cultures. Controls consisted of cultures transfected with MiniCas9V2 and a scrambled plasmid construct. After 48 h, samples were harvested for RNA isolation. The expression levels of each target gene were quantified by normalizing against the *Hprt* housekeeping gene.

**FIGURE 5:**
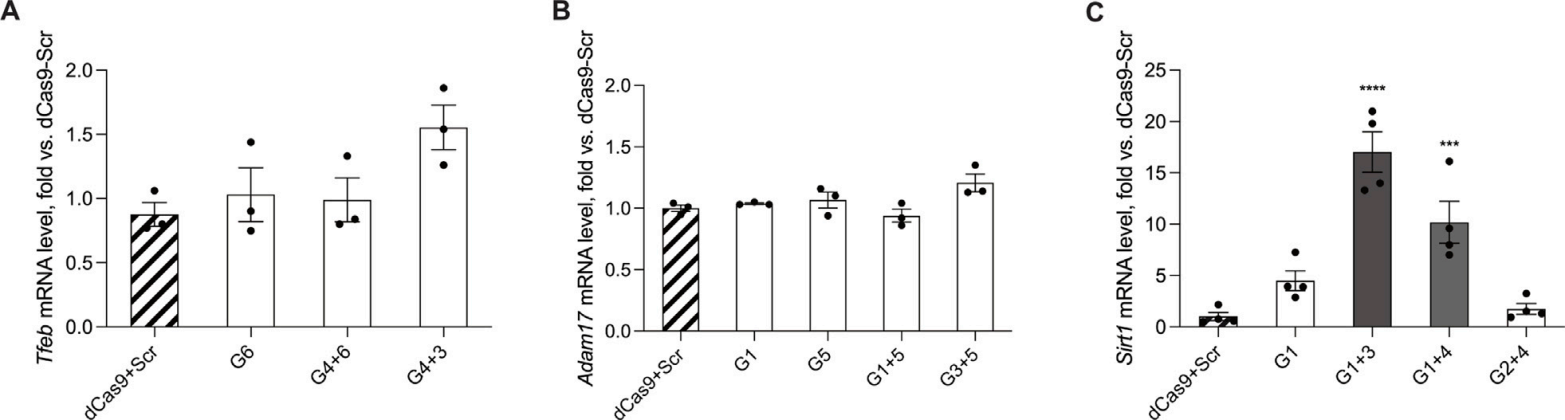
Quantification of endogenous *Tfeb*, *Adam17*, and *Sirt1* mRNA expression. Gene expression analysis of *Tfeb* **(A)**, *Adam17* **(B)**, and *Sirt1* **(C)** in Neuro2A cultures transfected with respective sgRNA combinations along with MiniCas9V2. Samples were normalized using mouse *Hprt* as a housekeeping control and quantified against the dCas9-Scr control. All values are presented as the mean ± SEM and quantified against a scrambled control (left, striped). For the histograms, bars with higher statistical significance are marked in darker shades of grey. For multiple comparisons, ***P ≤ 0.001, ****P ≤ 0.0001.

For the *Tfeb* promoter, a 1.5-fold increase in gene activation was observed in cultures transfected with sgRNA 4 + 3; however, this increase did not reach statistical significance (Figure 5A). In cells transfected with sgRNAs targeting the *Adam17* promoter, no significant increase in gene activity was detected (Figure 5B). Transfection with sgRNA 1 + 3 specific for *Sirt1* promoter revealed a statistically significant 17-fold increase in gene expression when compared to Scr baseline control (F (4,15) = 3.80), p < 0.05) (17.67 ± 7.04-fold) (Figure 5C). This also reflected a 4-fold higher activation of endogenous gene expression compared to the reporter-based screening assay (Figure 3C). In addition, a 10-fold increase in gene activation was detected in cells transfected with sgRNA 1 + 4.

This data demonstrates the gene activation capacity of selected sgRNA in promoter regions of interest. Importantly, however, it highlights the variability in activation potential between promoter regions, and accentuates the need for further investigation on the requirements for efficient gene upregulation.

## 4 Discussion

This study describes the design and validation of an experimental screening assay to systematically assess sgRNA potential for CRISPRa applications using viral vectors. Furthermore, a cloning workflow was created to further facilitate the omnibus cloning of sgRNA into LV and AAV. The screening assay was validated with a miniature dCas9 coupled to a VPR system (Maria et al., 2020) to systematically screen sgRNA for three different genes: *Tfeb, Sirt1* and *Adam17.* Activation of the promoter region proximal to the TSS was achieved in all genes tested, either by single sgRNA or dual sgRNA combinations. When transfected in Neuro2A cells, top single sgRNA or dual sgRNA combinations were able to significantly activate endogenous *Sirt1* transcription, while increase in *Tfeb* and *Adam17* activity remained ambiguous.

Golden Gate (Engler et al., 2008) is a versatile cloning method that enables scarless, multitiered omnibus assembly of up to 35 DNA fragments (Bird et al., 2022). It is commonly used in CRISPR studies to insert 20–21 bp double stranded DNA, encompassing the DNA binding region, into sgRNA expression cassettes already present in final expression constructs (Cong et al., 2013; Savell et al., 2019). This simple design, while optimal for single one-step sgRNA cloning, lacks the flexibility to switch or rearrange multiple sgRNA cassettes if needed. For this reason, Kabadi and colleagues developed a two-tier Golden Gate system where double-stranded DNA is first cloned and inserted into a plasmid containing one sgRNA expression cassette (Kabadi et al., 2014). A second Golden Gate reaction is then used to insert up to four different sgRNA cassettes into lentiviral transfer vectors containing an active Cas9 for gene editing. As this Golden Gate design uses different pol III promoters for each of the sgRNA cassettes, each short RNA will be expressed with different efficiencies (Goguen et al., 2021), potentially causing suboptimal activation, especially for multiplex activation. More recently, Savell and colleagues used Golden Gate to clone up to eight sgRNA cassettes in tandem, all driven by separate human U6 promoters, for multiplex CRISPRa using a dual lentiviral vector system (Savell et al., 2020). For our research needs, we required a flexible cloning workflow where sgRNAs would be first validated in a screening assay and subsequently cloned and inserted into an LV or AAV CRISPRa system. Although L3G0 cloning can assemble full sgRNA cassettes similarly to the systems described above, it differs in two key points. First, in the L3G0 workflow, the sgRNA cassettes are first screened before being cloned into viral vectors, in contrast with Savell et al., where the sgRNA are first cloned into an LV for individual screening and subsequently multiple sgRNA are re-cloned into a second LV for multiplex CRISPRa. Second, the L3G0 workflow combines up to eight sgRNA cassettes using multiple Golden Gate destination sites with the flexibility of subcloning these cassettes into LV and AAV. This workflow is designed to minimize the time and steps needed from initial sgRNA screening to final incorporation of validated sgRNA into viral vectors for *in vivo* or *ex vivo* gene therapy applications.

The design of effective sgRNAs is a key factor for successful CRISPRa applications (Kampmann, 2018; Casas-Mollano et al., 2020; Pandelakis et al., 2020; Liu et al., 2021). Commonly used tools such as CHOPCHOP (Labun et al., 2019) can be complemented further by machine learning to predict sgRNA function for CRISPRa applications (Horlbeck et al., 2016). However, designing and selecting sgRNA for CRISPRa using only bioinformatic tools has drawbacks. First, different Cas-proteins bind optimally at different promoter regions. For example, Cpf1 can activate genes by binding −600 to +400 from the TSS, in contrast with SpCas9, which uses +400 to 0 from the TSS (Gilbert et al., 2014; Tak et al., 2017; Chen et al., 2020). In addition, these predictive tools have been developed for SpCas9 (Horlbeck et al., 2016) and it is currently unclear how suitable these tools are for other Cas proteins. Hence, most CRISPRa studies either employ multiple sgRNA together without prior validation of activation potential (Kunii et al., 2018; Weltner et al., 2018; Choi et al., 2020) or select few sgRNA and assess their effectiveness experimentally (Cheng et al., 2013; Maeder et al., 2013; Savell et al., 2020; Schoger et al., 2020).

Using large cassettes containing multiple sgRNAs without any prior selection is suboptimal for gene therapy for several reasons. First, the inherent size constrains for cargo in viral vectors makes cassettes with excessive numbers of sgRNAs incompatible with viral vector delivery. Moreover, using multiple sgRNA increases the likelihood of off-target effects, and may also increase the chance of recombination events and deficient viral production (Casas-Mollano et al., 2020; Schoger et al., 2020). Importantly, multiple sgRNA usage does not guarantee robust gene activation and may result in reduced target gene expression, despite being common practice in CRISPRa applications (Cheng et al., 2013; Schoger et al., 2020).

Studies validating sgRNA often use RT-qPCR to test between two and seven sgRNA (Konermann et al., 2015; Kunii et al., 2018; Colasante et al., 2019; 2020; Savell et al., 2019; 2020; Choi et al., 2020). In contrast to these studies, we opted to screen sgRNA using a fluorescence-based assay. In addition to allowing a readout at protein level by quantifying transgene expression based on TdTomato, fluorescence-based methods are cost-effective and allow easy scaling-up of sgRNA screening. We started by developing fluorescence-based tools for the assessment of up to four sgRNAs (Maria et al., 2020). Moving forward, we optimized the assay conditions and integrated the assay into a cloning workflow, allowing us to screen up to 28 separate single- or combined sgRNAs in a 96-well plate format. This lets us systematically assess sgRNA efficacy throughout promoter regions relevant to MiniCas9V2.

Data from the experimental screening assay indicated that <50% of single sgRNAs resulted in significant promoter activation. This is in line with data from multiple *in vitro* and *in vivo* studies where not all genes were able to be activated with one sgRNA (Weltner et al., 2018; Savell et al., 2020; Schoger et al., 2020). In addition, our data show that similar to single sgRNAs, not all dual sgRNA combinations lead to activation. Again, this is in line with other studies that observed a lack of gene activation with several dual or multiple sgRNA combinations (Lin et al., 2015; Schoger et al., 2020). The maximum level of activation observed in the screening assay was also dependent on the promoter targeted, with *Tfeb* and *Sirt1* resulting in 4-to-5-fold activation, whereas *Adam17* led to 7-fold activation of their respective promoters. This suggests that not all individual sgRNA will cause gene activation and, given the preferentially antagonistic interaction between sgRNA, arbitrary selection without validation will likely result in false negatives. These points further highlight the importance of assessing such interactions before experimental applications. As such, the data from our study and previous literature suggest that the optimal number of sgRNAs required for robust CRISPRa activation is gene specific. Therefore, selecting and experimentally screening for the minimum amount of sgRNA/cRNA for CRISPRa is likely needed for optimal results.

When examining the effective relationships between sgRNA candidates, antagonistic interactions were observed more frequently than synergistic interactions. However, it remains important to assess the interactions between the respective sgRNA candidates along with the subsequent level of activation. As an example, in the screening assay for *Adam17,* sgRNA 4 displayed antagonism when coupled with either sgRNA 1 or sgRNA 5. When comparing sgRNA 1 + 4 to sgRNA 1 + 5 (Figure 3B), sgRNA 1 + 4 displays a complete lack of activation along with a strong antagonistic interaction. Looking further, sgRNA 1 alone has a statistically significant activation which appears nullified when combined with sgRNA 4. This suggests that the antagonistic response of sgRNA 4 is enough to abolish the otherwise effective candidate sgRNA 1. However, in sgRNA 1 + 5, the detected antagonism coupled with a strong activation suggests that the gene may already operate at a maximum level of activity due to sgRNA 5 and is not nullified by the seemingly antagonistic interaction with sgRNA 1. These observations underline the importance of considering sgRNA antagonism in concert with the resulting gene activity response.

The data generated by the screening assay was also used to correlate sgRNA activation with TSS and TFBS present in the promoter region. Only *Sirt1* showed both an inverse correlation between activation and number of TFBS overlapping the sgRNA binding sequence and a correlation between activation and number of TFBS within 50 bp of sgRNA binding. Although this dataset is limited, we were not able to discern any guidelines to improve *in silico* sgRNA design for CRISPRa using *S. aureus* Cas9. The majority of other studies developing *in silico* prediction and optimization tools for sgRNA design for CRISPRa are based on *S. pyogenes* Cas9 (Gilbert et al., 2014; Bergenholm et al., 2021; Yang et al., 2023). The lack of published design tools for *S. aureus* Cas9 CRISPRa sgRNA, combined with the analysis of data generated by the screening assay, suggests that the activation potency of sgRNAs need to be screened experimentally.

From inception, the screening assay was designed to test sgRNAs/cRNAs for any gene or Cas protein. However, the current iteration of the screening assay has limitations. As the assay is plasmid-based, it does not consider gene and cell-specific epigenetics. For this reason, top-selected sgRNAs may need to be further tested on endogenous genes to fully determine their potential for gene activation. Additionally, investigation of the epigenomic landscape of targets of interest using bioinformatic approaches may be helpful in more closely predicting the outcome of these epigenetic gene regulation tools. Nevertheless, it remains unclear what impact epigenetic modifications have on CRISPRa as effective sgRNAs can activate the same genes across different cell types, whether in culture or *in vivo* (Cheng et al., 2013; Maeder et al., 2013; Chavez et al., 2015; Kiani et al., 2015; Vora et al., 2018; Zhou et al., 2018; Colasante et al., 2019; Colasante et al., 2020; Matharu et al., 2019; Savell et al., 2019; Savell et al., 2020; Maria et al., 2020; Schoger et al., 2020; Giehrl-Schwab et al., 2022).

In the *Adam17* screening assay, we observed similarly significant levels of gene activation with sgRNA 5, sgRNA 1 + 5, and sgRNA 3 + 5. Interestingly, at the endogenous mRNA level, no significant increase in gene expression was detected after transfection with these sgRNA combinations. A similar trend was observed in the *Tfeb* assay, where sgRNA 6, sgRNA 4 + 6, and sgRNA 4 + 3 all showed significant upregulation, but only sgRNA 4 + 3 resulted in a near-statistically significant activation at the mRNA level. In contrast, when targeting *Sirt1,* a significant increase in endogenous mRNA was observed, particularly in cultures transfected with sgRNA 1 + 3, which led to a 17-fold increase in gene expression compared to the control. This reflected a 4-fold higher activation level than predicted by the screening. These results suggest a discrepancy in sgRNA efficacy when targeting a fully accessible promoter, such as that from a transfected plasmid, compared to an endogenous target. Additionally, sgRNAs may act more effectively in reporter models with a high number of available transgene copies, compared to endogenous genes where only two target copies are available for regulation, therefore promoting a different kinetic model (Duarte et al., 2023). It is therefore important to consider that upregulation of therapeutic targets generally entails activating genes that have minimal constitutive expression. Thus, in some genes, the activation levels may not be dramatically increased, unlike proof-of-concept genes that may be more easily targeted and regulated.

Given the variation in expression ceilings for different genes, a functional readout may be required in addition to bulk expression analysis. In Neuro2A cultures, lentiviral transduction of *Tfeb, Adam17, or Sirt1* sgRNA constructs in combination with MiniCas9V2 failed to increase endogenous gene expression (data not shown). However, a near-significant increase in *Tfeb* activity was observed when sgRNA 4 + 3 were co-transfected, along with the previously noted increase in *Sirt1* expression upon co-transfection of sgRNA 1 + 3 or sgRNA 1 + 4. No significant increase in *Adam17* expression was observed under any condition (Figure 5). This suggests a discrepancy in assessing functional response between transfection- and transduction-based methods and may be important when determining the most reliable approach to assess the functional readouts of different genes of interest (Duarte et al., 2023). It may also indicate differences in promoter accessibility and propensity for activation using this particular gene regulation system.

In this study, we focused our efforts on assessing gene regulation specifically at the genetic level. However, post-transcriptional and post-translational effects of gene activation may add additional divergence between targets of interest. For instance, Tfeb’s nature as a transcription factor with broad effects on cellular homeostasis and the autophagy response (Napolitano and Ballabio, 2016; Chen et al., 2021) may contribute to making a difficult target to regulate epigenetically. Additionally, given the strict regulation of Tfeb under healthy cellular conditions, the functional readout of *Tfeb* activation may be more efficiently studied in disease models of protein aggregation to determine potential therapeutic effects. Indeed, information on the transcriptional regulation of *Tfeb*, particularly at an epigenetic level, is limited. Alternatively, combining epigenetic regulation of the *Tfeb* promoter region with additional modifications of the chromatin state, or inducing Tfeb activation at the protein level, may yield more favorable effects on functionality.

In summary, we describe a systematic experimental approach for screening sgRNA/cRNA for CRISPRa studies. We identify multiple sgRNA combinations capable of upregulating gene expression in several genetic targets. Additionally, we highlight the need for further studies on the optimization of gene activation using CRISPRa. The multiplexing potential of this workflow could aid in accelerating and streamlining the development of future CRISPRa-focused applications.

## Data Availability

The raw data supporting the conclusions of this article will be made available by the authors, without undue reservation.
