# Supplementary material for: A systematic screening assay identifies efficient small guide RNAs for CRISPR activation
Source: Front Bioeng Biotechnol. 2025 Jan 23;13:1336313. doi: 10.3389/fbioe.2025.1336313 (PMC11799263; doi:10.3389/fbioe.2025.1336313)
Supplement: Supplementary file 1 [file Image1.pdf]

# A systematic screening assay identifies efficient small guide RNAs for CRISPR activation

**Elin Arvidsson[1], Diana Duarte Lobo[2, 3], Ermelinda Sabarese[1], Fabio Duarte[1], Rui Jorge Nobre[2, 3, 4], Luis Quintino[1], Cecilia Lundberg[1*]**

[1] CNS Gene Therapy, Department of Experimental Medical Sciences, Lund University, Lund, Sweden

[2] Molecular Therapy of Brain Disorders Group, CNC - Center for Neuroscience and Cell Biology of Coimbra, University of Coimbra, Coimbra, Portugal

[3] Institute for Interdisciplinary Research, University of Coimbra, Coimbra, Portugal

[4] ViraVector – Viral Vector for Gene Transfer Core Facility, University of Coimbra, Coimbra, Portugal

*** Correspondence:**
Cecilia Lundberg
cecilia.lundberg@med.lu.se

# Supplementary Material

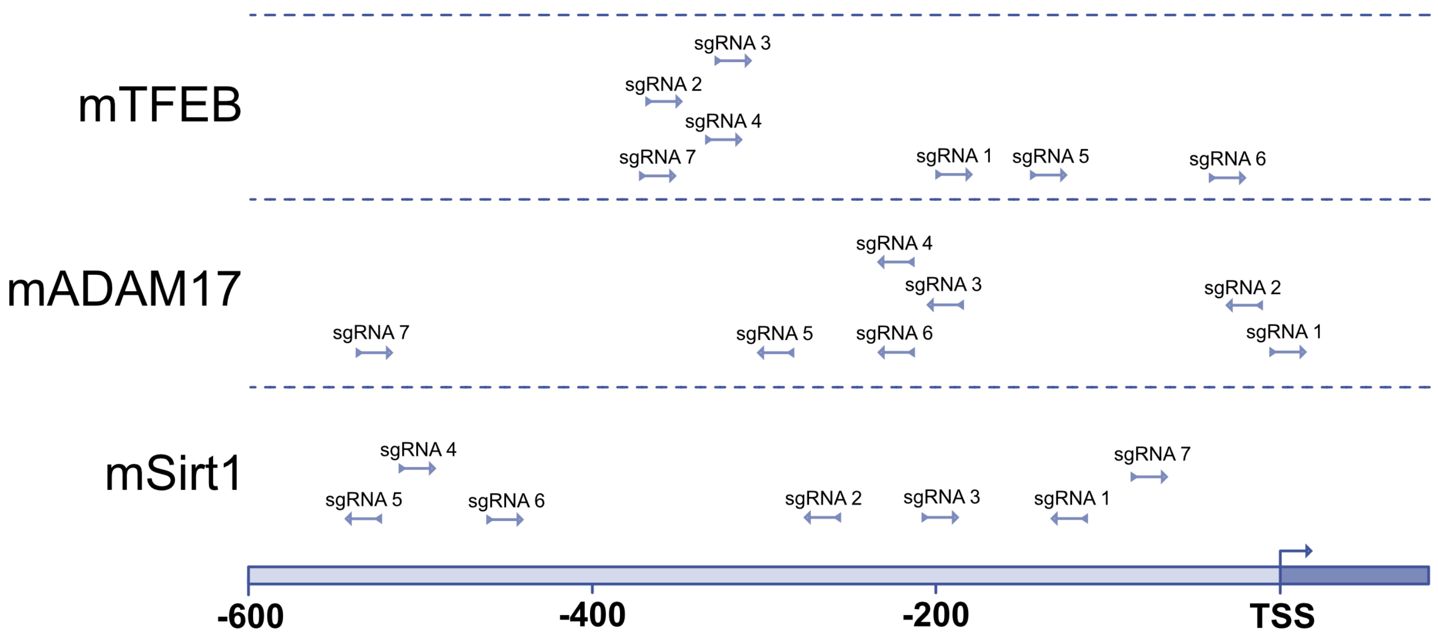


**Supplementary figure 1: sgRNA sequences relative to transcriptional start site.**

sgRNA 1 to 7 for mouse Tfeb, mouse Adam17 and mouse Sirt1 illustrated relative to the transcriptional start site (TSS) for each of their respective genes.

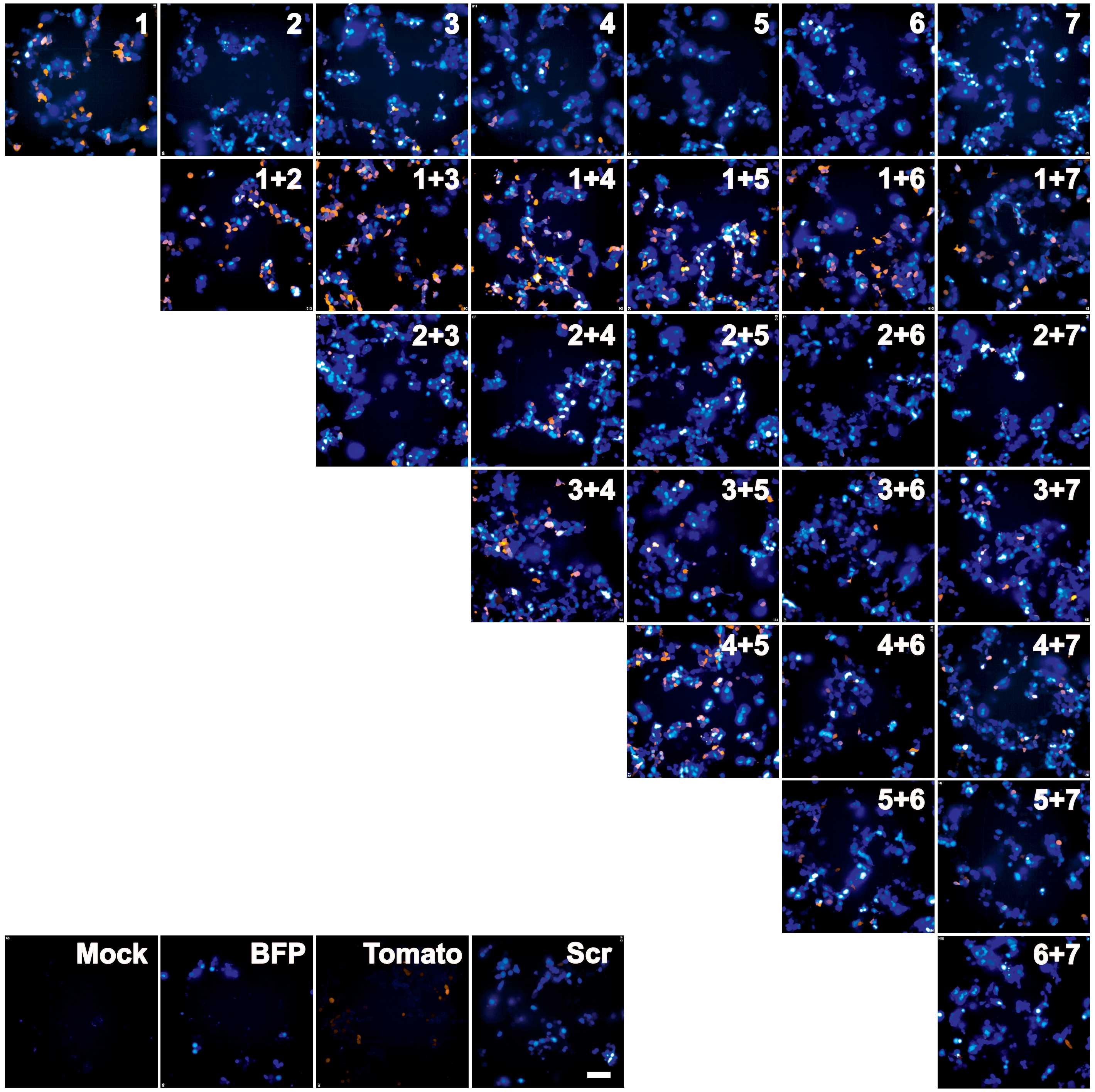


**Supplementary figure 2: Fluorescence images of the screening assay using mouse Sirt1.**

293T cells were transfected with pDPL0 expressing sgRNA, reporter constructs expressing TdTomato, MiniCas9V2 and BFP. Forty-eight hours after transfection, the cells were fixed and the TdTomato and BFP fluorescence were measured. Each number denotes an sgRNA. Mock - mock transfected cells. BFP - cells transfected with BFP and MiniCas9V2. Tomato - cells transfected with TdTomato reporter and MiniCas9V2. Scr - cells transfected with Scramble sgRNA, MiniCas9V2, TdTomato reporter and BFP. Scale bar -100 µm.
